# Miniaturisation of a laser-scribed graphene electrode enables analyte detection at ultra-low concentrations

**DOI:** 10.1039/d6ra01797e

**Published:** 2026-04-21

**Authors:** Sarojini Prusty, Shubham Upadhye, Pranati Nayak

**Affiliations:** a Department of Engineering and Materials Physics, Institute of Chemical Technology-Indian Oil Odisha Campus Bhubaneswar 751013 India pranati2008@gmail.com p.nayak@iocb.ictmumbai.edu.in

## Abstract

The detection of analytes at ultra-low concentrations using macroelectrodes is challenging because large capacitive currents mask faradaic currents. Reducing the sensing geometry of electrodes to the micro- or nanoscale significantly boosts the faradaic current relative to the capacitive current. Although lower-dimensional electrodes perform exceptionally well, fabricating micro-sized sensors on a large scale remains a major challenge. In this work, we introduce a simple, scalable fabrication method for creating graphene-based miniaturized electrodes (∼200 µm width) *via* direct laser writing using a CO_2_ laser with a wavelength of 10.6 µm. The resulting LSG (laser-scribed graphene) electrode features a high specific surface area, excellent electrical conductivity, and tunable surface chemistry, making it highly suitable for electrochemical sensing applications. We evaluated the electrochemical performance of the electrode for dopamine detection over a broad dynamic concentration range from 0.167 µM to 18.634 µM. We achieved a sensitivity of ∼0.4 ± 0.013 µA µM^−1^ cm^−2^, a limit of detection (LOD) of 0.41 µM, and an average response time of ∼0.1 s. Given their simple and scalable fabrication process, low cost, and efficient detection capabilities, LSG-based miniaturized electrodes are highly recommended as an effective platform for the monitoring of neurotransmitters in biomedical and clinical settings.

## Abstract 2

Miniaturized electrode is more efficient in detecting analyte compared to its macro counterpart.

## Graphical abstract

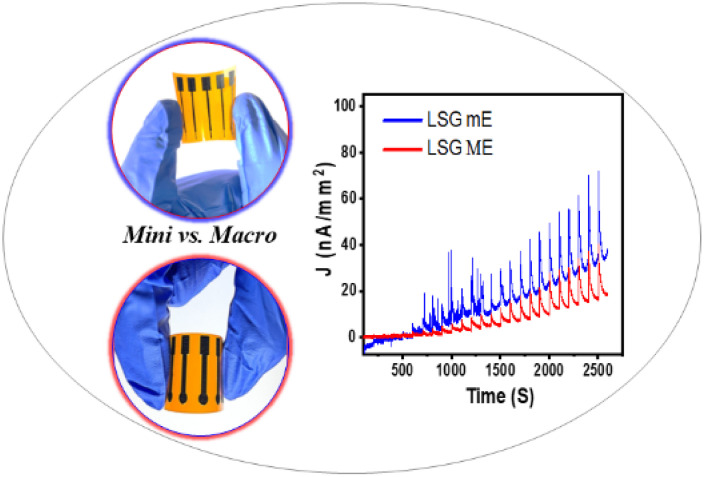

## Introduction

Electrode miniaturisation has gained popularity in electroanalytical device fabrication, as it enhances electrochemical properties and cuts device costs by reducing their size.^1^ While nano-electrodes are smaller, microelectrodes are especially favoured for their ease of production and handling.^2,3^ They offer high spatial resolution and sensitivity by decreasing capacitive background currents, improving mass transport, and minimizing ohmic drop.^4,5^ Unlike macroelectrodes, in which linear diffusion dominates, miniaturized electrodes with microdimensions enable convergent or radial diffusion, leading to faster kinetics and better detection of low analyte concentrations.^5^ Although gold and platinum are more commonly used in microelectrode fabrication, carbon-based microelectrodes present several advantages, such as a wide potential window, controllable surface activity through functional groups, and porosity.^6^ Notably, graphene-based microelectrodes attain low detection limits and high sensitivity due to their rapid charge transfer and large electrochemically active surface area.^7,8^ The adjustable surface porosity and high electron conductivity of multilayer graphene improve ion access and charge transport. Notably, the surface roughness of graphene edges makes them rich in electrochemically active sites, enabling more accurate analyte detection.^9^

Despite these advantages, the key challenge in miniaturising graphene-based electrodes in electro analytical devices is the lack of large-scale production methods and high costs.^10^ Current methods for fabricating graphene-based microelectrodes include photolithography, screen printing, spray coating, and 3D printing techniques.^11–13^ Although these methods have successfully produced graphene microelectrodes and arrays on a large scale, they are mostly limited to laboratory-scale, multistep wet-chemistry processes. Furthermore, the fabrication process often requires the use of polymeric binders and additives, along with post-drying treatments, which significantly reduce key properties such as high specific surface area and conductivity due to sheet restacking. To address this, the development of binder-free, porous, and durable graphene electrodes of miniaturised size is essential.

In this context, direct laser writing (DLW) has shown promise, as it offers a scalable, easy-to-fabricate, low-cost method for creating laser-scribed graphene (LSG) electrodes with high conductivity, a large specific surface area, and numerous edge-plane sites.^14^ DLW is a computer-aided scribing technique capable of producing user-defined patterns with dimensions down to the laser spot size of 200 microns.^15^ In the past, LSG-based electrodes have demonstrated potential for detecting biomarkers such as glucose, dopamine, cholesterol, and others, with high sensitivity and selectivity due to their appealing properties, such as a large potential window, charge transfer rate, and conductivity.^16–20^ It is demonstrated that LSG can be fabricated down to a laser spot size under the optimised conditions.^21^ Owing to the alluring electrochemical properties of LSG and the viability of DLW for device miniaturisation, it is interesting to investigate the electrochemical sensing performance of the miniaturised LSG electrodes. Herein, we have designed LSG-based miniaturised devices with a microdimensional (200 µm width) working area, explored their electrochemistry compared to that of macro-sized electrodes, and assessed their viability for sensing neurotransmitters at ultralow concentrations.

## Experimental

### Materials and methods

Hexaammineruthenium chloride [Ru(NH_3_)_6_Cl_3_], potassium chloride (KCl), sodium phosphate dibasic (Na_2_HPO_4_), sodium phosphate monobasic (NaH_2_PO_4_), dopamine hydrochloride (DA), and potassium hexacyanoferrate K_4_[Fe(CN)_6_]·3H_2_O were procured from Sigma-Aldrich and used as received. Polyimide (PI) sheets with a thickness of 125 µm were obtained from DuPont, USA. Conducting silver paste was purchased from Ted Pella, UK. 0.1 M phosphate buffer solution (PBS) was prepared by mixing Na_2_HPO_4_ and NaH_2_PO_4_ and adjusting the pH to 7. All the electrolytes and analytes for electrochemical measurements were prepared using ultrapure water (Millipore water with a resistivity of 18.2 MΩ cm from Merck, MilliQ) before each electrochemical measurement. For electrode surface passivation, SYLGARD 184 silicone elastomer (Dow Corning) was used by mixing the base elastomer with the curing agent in a 10 : 1 ratio, followed by degassing under vacuum to remove air bubbles before use.

### Electrode fabrication

**Fig. 1:**
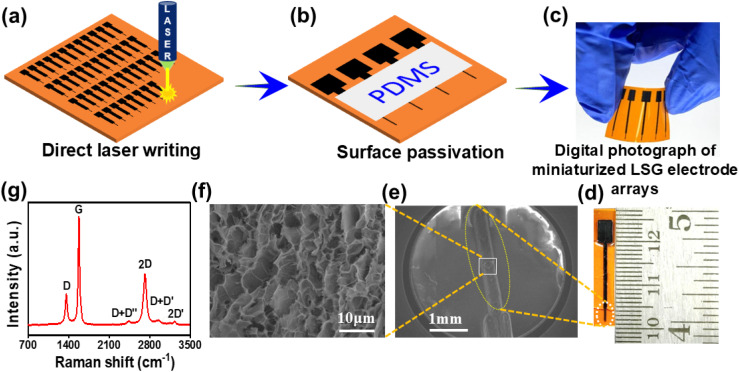
Schematic illustration of the fabrication of LSG mE patterns on a PI sheet. (a) Fabrication of an array of electrodes on a PI sheet by direct laser writing. (b) Selective surface passivation of the LSG mEs by PDMS. Digital photographs of (c) a patterned electrode array and (d) a single electrode. (e) Projection display of the FE-SEM image of the microdimensional electrode area. (f) SEM image of the electrode surface at high magnification. (g) Raman spectra of LSG.

To fabricate LSG miniaturised electrodes (LSG mE) *via* direct laser writing, a CO_2_ laser (75 W, Universal Laser System) was used with a wavelength of 10.6 µm and a pulse duration of approximately 14 µs.^15^ The laser parameters, such as speed, ppi (pulses per inch), and the *z* distance (distance between the laser beam and the PI sheet), were optimized to 5.8 cm, 1000 and 2 mm, respectively, to produce highly conducting graphitic carbon. The DLW experiments were performed under ambient conditions using CorelDRAW software to design the electrode pattern. To scribe patterns of the minimum possible width, a one-line scribing technique was implemented (Fig. 1). The overall electrode dimensions were 2.5 cm × 5 mm with a 200 µm × 3 mm working electrode area for convenient use in an electrochemical cell. The geometrical area of the working electrode was defined by selectively passivating the rest of the area of the electrode with PDMS, followed by drying in a vacuum oven at 80 °C for 30 minutes. To ensure good electrical contact, copper tape was attached to the electrode with conductive silver paint. For comparison, LSG macro electrodes (LSG ME, 3 mm diameter WE area) were fabricated using the same laser settings, as shown in Fig. S1, SI. In addition, carbon fibre microelectrodes were prepared from commercial carbon fibres using an indigenously developed method.^22^

### Electrochemical characterization

All electrochemical measurements were performed using an Autolab electrochemical workstation (Metrohm, Netherlands) in a 25 mL glass cell, controlled by NOVA 2.1.5 software. A standard three-electrode system was employed, consisting of an Ag/AgCl (3 M KCl) reference electrode, a platinum wire counter electrode (supplied by CH Instruments, Austin), and a fabricated LSG working electrode. All experiments were conducted under thermostatic conditions (25 °C) inside a Faraday cage. Cyclic voltammograms were recorded under different potential windows for various redox mediators at different scan rates. The chronoamperometric technique was used to determine the analyte concentration at specific times. The electrolyte was de-aerated before each measurement, and data analysis was performed using the software Origin.

## Results and discussion

### Design and functional characterization of electrodes

The schematics show the step-by-step process of the fabrication and characterisation of LSG mEs. DLW allows large-scale fabrication of LSG mEs over a PI sheet, as depicted in Fig. 1a. The micro-pattern (200 µm × 3 mm) was scribed at the edge of a 3 cm length LSG electrode for experimental convenience. The laser spot size of 200 µm limits scribing lines to smaller dimensions. The obtained micro patterns have a typical thickness of ∼33 µm, as can be seen in the cross-sectional FESEM image in Fig. S2, SI. The as-fabricated electrode was selectively passivated using PDMS (Fig. 1b) in order to define the area of the WE. Digital images of the electrode arrays and a single LSG mE are shown in Fig. 1c and d, respectively.

The morphology of the LSG mE was characterised by FESEM, as shown in Fig. 1e and f. The LSG surface appears to be highly porous and rich in edge plane sites that provide abundant reactive sites for electrochemical reactions. Additionally, the 3D morphology enables a large electrochemically active surface area. The nature of the carbon produced *via* DLW of the Kapton (PI) sheet was further analysed using Raman spectroscopy. Fig. 1f shows a typical Raman spectrum exhibiting the dominant characteristic bands, namely, the D, G, and 2D bands, corresponding to graphitic carbon.^23^ The disordered D band appears at about 1360 cm^−1^, indicating the presence of structural defects or stacking disorder between layers.^23^ The G band at around 1578 cm^−1^ corresponds to the E_2g_ vibrational mode of graphitic carbon and is considered to be the key mode (longitudinal optical phonon mode) in graphene, which suggests a planar configuration of sp^2^ hybridised carbon atoms.^24^ The 2D band is regarded as the second-order overtone of the D band, resulting from a two-phonon lattice vibrational process, and indicates the presence of few-layer graphene structures. Along with these, weaker vibrational peaks corresponding to D + D′, D + D″, and 2D′ are also present, thus indicating the presence of sp^2^ hybridised graphitic carbon in the LSG matrix.

### Electrochemical performances

**Fig. 2:**
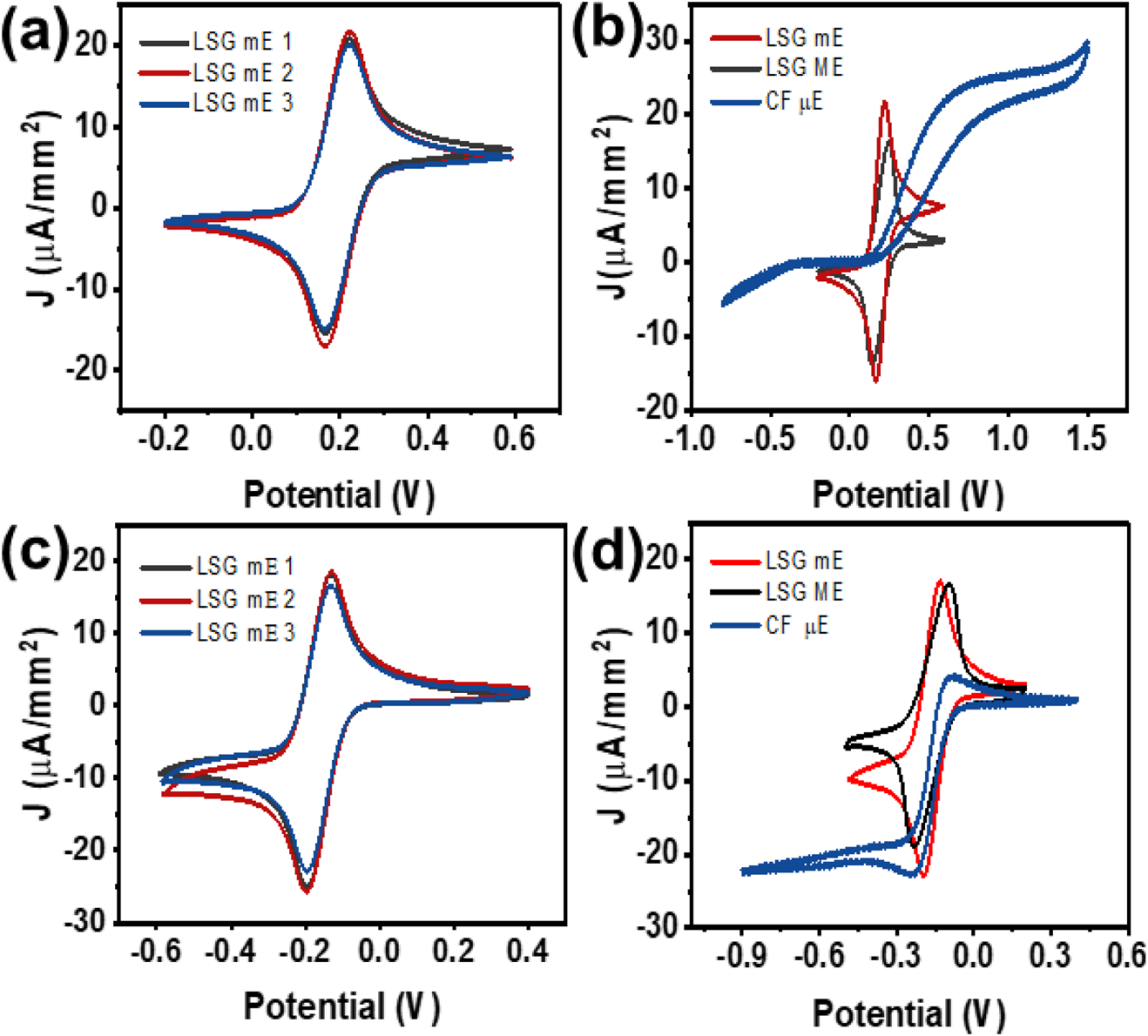
Comparison of the CV plots for (a) three different LSG mEs and (b) an LSG mE, LSG ME, and a carbon fiber µE in 5 mM [(Fe(CN)_6_)]^3−/4−^. (c) Comparison of the CV plots for three different LSG mEs and (d) an LSG mE, LSG ME, and a carbon fiber µE in 5 mM [Ru(NH_3_)_6_]^3+/2+^ with 0.1 M KCL solution at 50 mV s^−1^.

The electrochemical performance of the LSG mE was evaluated through a series of cyclic voltammetry measurements using both inner-sphere ([(Fe(CN)_6_)]^3−/4−^) and outer-sphere ([Ru(NH_3_)_6_]^3+/2+^) redox mediators, each at a concentration of 5 mM in 0.1 M KCl as the supporting electrolyte. The voltammogram shows reversible redox peaks with an average peak-to-peak separation (Δ*E*_p_) of about 54.467 mV, indicating efficient electron transfer in the LSG mE (Fig. 2a).^25,26^ CV experiments were performed for four different LSG mEs at different scan rates, as shown in Fig. S3. As observed, the LSG mEs produced *via* DLW are highly reproducible and reliable, as confirmed by the consistent peak currents across three different electrodes under identical experimental conditions (Fig. 2a). Additionally, an increased current density at a smaller Δ*E*_p_ value (∼56.1 mV) is observed for the LSG mE compared to the LSG ME (Δ*E*_p_ ∼107.4 mV) and CF µE (Δ*E*_p_ ∼691 mV) (Fig. 2b). Repeated measurements were performed for the carbon fiber µEs as well, as shown in Fig. S4. CVs were conducted for electrodes with different geometries (Fig. S5a–c, SI) and of various sizes with the same geometry (Fig. S5d–g, SI). The Δ*E*_p_ was observed to increase with increasing geometric area (Fig. S5c), indicating a decrease in the charge transfer rate. Additionally, for electrodes with different sizes but the same geometry, a very similar increasing trend in Δ*E*_p_ is observed (Fig. S5g) with increasing size. Hence, it is evident that the electrochemical performance depends on the size and geometry of the electrodes. This is likely due to the larger diffusional flux in the small geometrical area of miniaturized electrodes compared to macroelectrodes, resulting in faster charge transfer.^27^ A very similar feature is observed for the outer-sphere redox probe, [Ru(NH_3_)_6_]^3+/2+^, with an Δ*E*_p_ value of about 63 mV with high repeatability (Fig. 2c). The observed Δ*E*_p_ is much lower compared to that for the LSG ME (Δ*E*_p_ ∼134.3 mV) and CF µE (Δ*E*_p_ ∼138.3 mV); however, no significant change in peak current is observed due to the outer-sphere nature of the redox probe (Fig. 2d).^28^ Furthermore, these values outperform those of other carbon electrodes such as glassy carbon, graphite, and graphene-based systems, indicating that LSG mEs are advantageous for electrochemical sensing applications.^29,30^

**Fig. 3:**
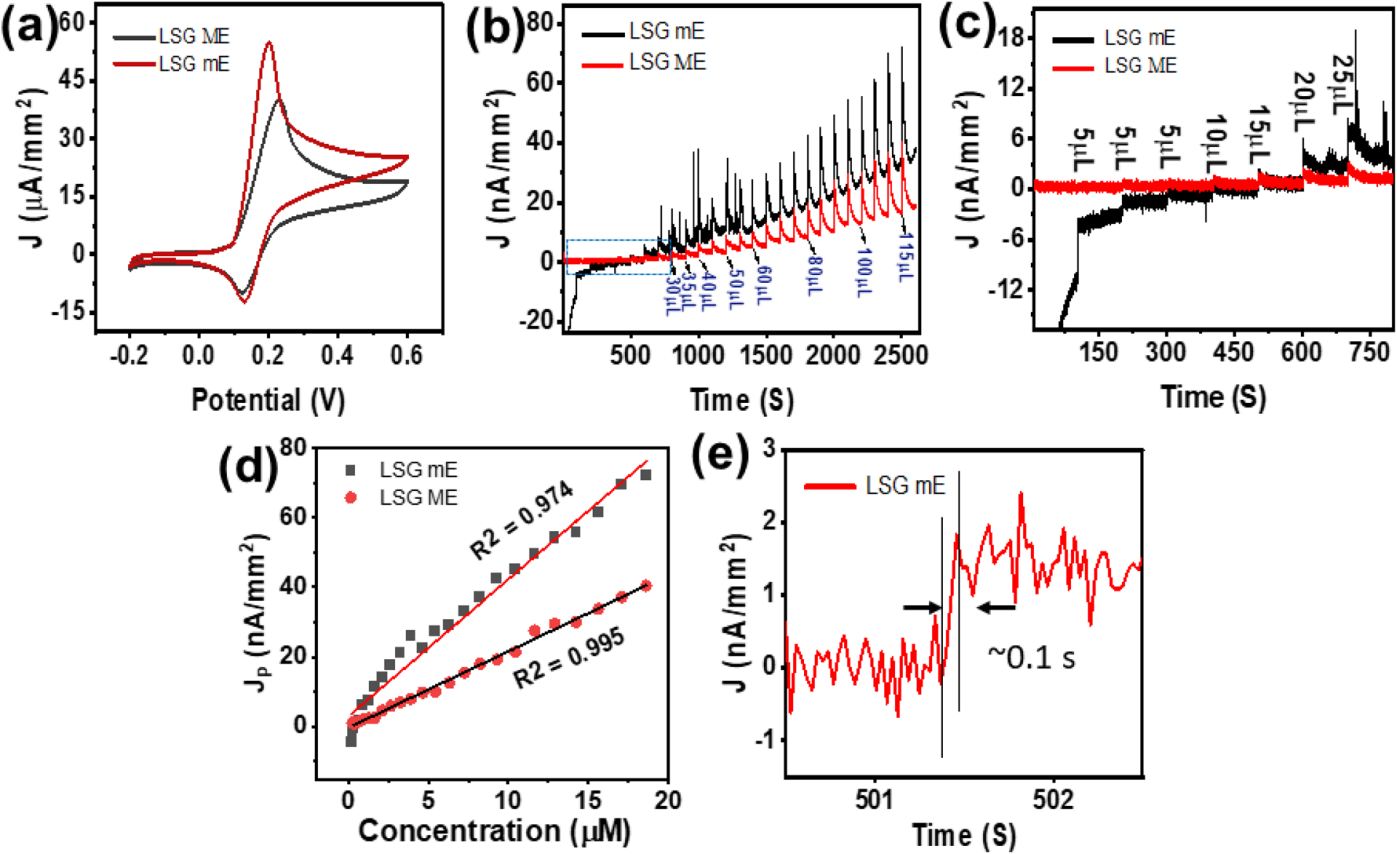
Comparison of the CV plots of (a) LSG mE and LSG ME for 5 mM DA in 0.1 M PBS solution at 100 mV s^−1^. (b) Amperometric response of LSG mE and LSG ME to dopamine concentrations from 0.167 µM to 18.634 µM in 0.1 M PBS (pH 7.4). (c) Expansion of the selected area of the amperogram in (b). (d) Linear calibration plot based on part (b). (e) Response time of the LSG mE to dopamine.

Electrode miniaturisation improves electron transfer kinetics and enables detection at ultra-low analyte concentrations by reducing capacitive background current and the ohmic drop.^31^ We validated this concept using dopamine (DA), a promising neurotransmitter in the human brain. Fig. 3a shows a comparison of the CV plots of LSG mE and LSG ME at a scan rate of 100 mV s^−1^. As observed, the dopamine oxidation potential is much lower, and the oxidation current is significantly higher for the LSG mE (∼54.83 µA mm^−2^) compared to LSG ME (40.121 µA mm^−2^), indicating more efficient electron transfer in the LSG mE. This is most likely due to enhanced radial diffusion at the microelectrode surface.^32^ The CV plots at different scan rates for three different LSG mEs and LSG MEs are shown in Fig. S6. To further analyse the efficacy of the LSG mEs, we conducted chronoamperometry analysis for both LSG MEs and LSG mEs for the addition of different concentrations of dopamine (Fig. 3b). As observed, the amperometric response for the LSG mEs upon the successive addition of dopamine is much higher compared to that of the LSG MEs. Fig. 3c shows the expansion of the selected area for low dopamine concentration. The plot shows linearity from 0.167 µM to 18.634 µM, as depicted in Fig. 3d. Notably, the LSG mE achieved a sensitivity of 0.4 ± 0.013 µA µM^−1^ cm^−2^ at a low limit of detection (LoD: 0.41 µM), which exceeds that of the macroelectrode (sensitivity: 0.215 ± 0.002 µA µM^−1^ cm^−2^, LoD: 0.95 µM). Additionally, the average response time was found to be ∼0.1 s, which further confirms the advantages of the miniaturized electrode architecture for sensitive and rapid dopamine detection. The average response time (ART) was calculated, and the LSG mE had a lower ART than the macroelectrode.

**Fig. 4:**
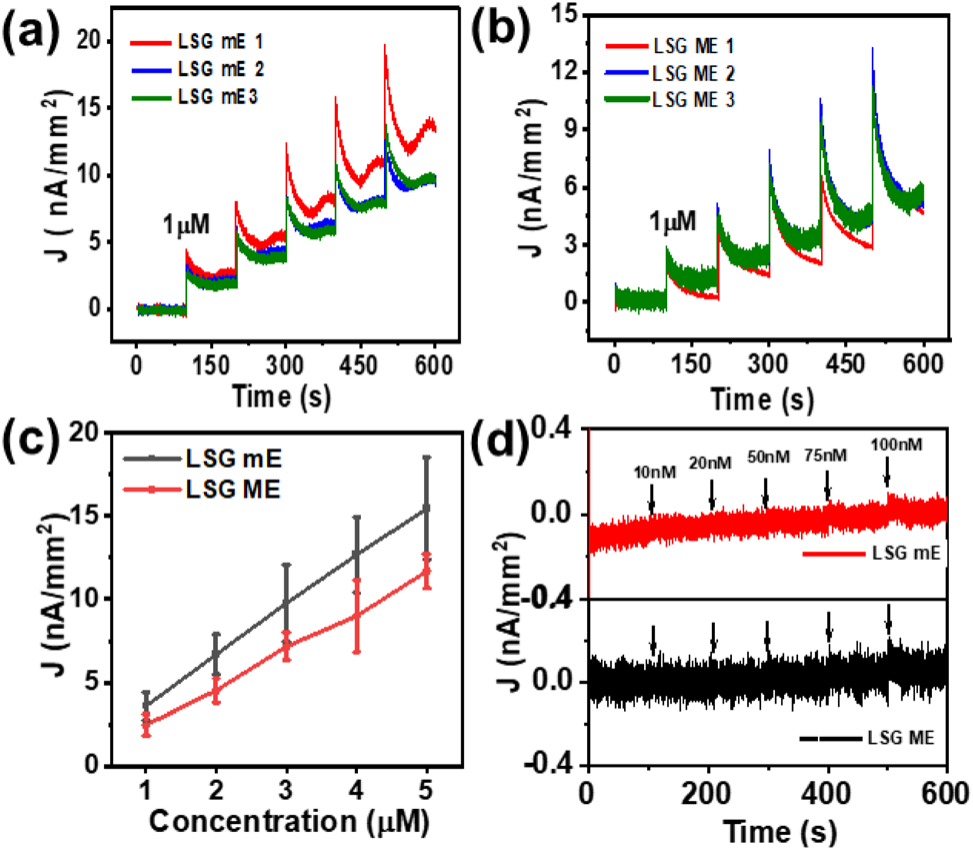
Amperometric response of (a) LSG mEs and (b) LSG MEs to successive additions of 1 µM dopamine. (c) Comparison of the linear calibration plots derived from (a) and (b). (d) Chronoamperogram (expansion of selected area) for the addition of ultra-low concentrations of DA.

Further chronoamperometry was performed under identical conditions for three different LSG mEs and LSG MEs (Fig. 4a and b) with the successive addition of 1 µM of analyte. The chronoamperometric plot showed that the LSG mEs exhibited a higher peak current density compared to the LSG MEs with the addition of a concentration of 1 µM. Along with this, the electrodes showed good reproducibility for each addition, and the average current value plotted against concentration showed linear behavior (Fig. 4c). This difference can be attributed to its miniaturised structure, enhanced electron transfer, and improved mass transport *via* radial diffusion, compared to those of a larger-sized LSG macroelectrode.^33^ The calibration plots for three different LSG mEs and LSG MEs are shown in Fig. S7. To explore the ability of the LSG mEs to detect ultra-low concentrations of analyte, we conducted amperometry for the addition of ultralow analyte concentrations from 10 nM to 100 nM. As observed from Fig. 4d, for the LSG mEs, a detectable current change appears at a concentration of 75 nM; however, for the LSG MEs, the current change is insignificant until 100 nM. The LSG mEs exhibit lower background noise and sharper signal currents compared to the LSG MEs, demonstrating superior resolution. This is due to the visibly larger baseline current width of the LSG MEs compared to the LSG mEs owing to their larger dimensions. This significantly suppresses the faradaic current due to analyte oxidation at low concentration. The original chronoamperogram is shown in Fig. S8, SI. These observations clearly indicate that the LSG mEs are more capable of detecting ultralow concentrations of analyte compared to LSG MEs. These findings confirm the improved electrochemical performance of LSG mEs for sensitive analyte detection and indicate that the LSG mE outperforms the LSG MEs in terms of sensitivity, current density, and LOD, making it the optimal choice for ultra-sensitive dopamine detection in electrochemical sensing applications.

## Conclusion

We demonstrated the successful fabrication of graphene-based miniaturized electrode arrays using direct laser writing on commercial polyimide. The method is simple and enables the large-scale production of miniaturized electrodes in a single step. We observed that miniaturisation of the working electrode from macro-to microdimensions improves the electrochemical performance. Compared to macrodimensional LSG electrodes, the microdevice showed improved potential for detecting dopamine over a wide linear range from 0.167 µM to 18.634 µM with a sensitivity of 0.4 ± 0.013 µA µM^−1^ cm^−2^. In addition, the sensor showed a response time of ∼0.1 s, a detection limit of 0.41 µM and good reproducibility. These results indicate that size miniaturisation reduces costs and significantly enhances performance.

## Author contributions

Sarojini Prusty: conducted the experiment, data analysis and interpretation, and manuscript writing; Shubham Upadhye: material characterization; Pranati Nayak: conceptualization, manuscript review & editing, supervision.

## Conflicts of interest

The authors confirm that there are no conflicts to declare.

## Supplementary Material

RA-016-D6RA01797E-s001

## Data Availability

The authors declare that the data supporting this manuscript's findings can be found in the supplementary information (SI). The raw data are available from the corresponding author upon request. Supplementary information is available. See DOI: https://doi.org/10.1039/d6ra01797e.

## References

[cit1] Da Silva P. F., Ribeiro T. S., Gomes B. F., Da Silva G. T. d. S. T., Lobo C. M. S., Carmo M., Ribeiro C., Filho R. B., Roth C., Colnago L. A. (2022). Anal. Chem..

[cit2] Akinoglu E. M., Kätelhön E., Pampel J., Ban Z., Antonietti M., Compton R. G., Giersig M. (2018). Carbon.

[cit3] Soleymani L., Li F. (2017). ACS Sens..

[cit4] Forster R. J. (1994). Chem. Soc. Rev..

[cit5] Menshykau D., Del Campo F. J., Muñoz F. X., Compton R. G. (2009). Sens. Actuators, B.

[cit6] Muqaddas S., Javed M., Nadeem S., Asghar M. A., Haider A., Ahmad M., Ashraf A. R., Nazir A., Iqbal M., Alwadai N., Ahmad A., Ali A. (2023). ACS Omega.

[cit7] Viana D., Walston S. T., Masvidal-Codina E., Illa X., Rodríguez-Meana B., Del Valle J., Hayward A., Dodd A., Loret T., Prats-Alfonso E., De La Oliva N., Palma M., Del Corro E., Del Pilar Bernicola M., Rodríguez-Lucas E., Gener T., De La Cruz J. M., Torres-Miranda M., Duvan F. T., Ria N., Sperling J., Martí-Sánchez S., Spadaro M. C., Hébert C., Savage S., Arbiol J., Guimerà-Brunet A., Puig M. V., Yvert B., Navarro X., Kostarelos K., Garrido J. A. (2024). Nat. Nanotechnol..

[cit8] Niaraki A., McNamara M. C., Montazami R., Hashemi N. N. (2022). ACS Appl. Bio Mater..

[cit9] j Velický M., Toth P. S., Woods C. R., Novoselov K. S., Dryfe R. A. W. (2019). J. Phys. Chem. C.

[cit10] Li F., Xue M., Ma X., Zhang M., Cao T. (2011). Anal. Chem..

[cit11] Hyun W. J., Secor E. B., Hersam M. C., Frisbie C. D., Francis L. F. (2015). Adv. Mater..

[cit12] Li Y., Zhao Y., Ruocco A., Wang M., Li B., Akhavan S. (2025). ACS Appl. Mater. Interfaces.

[cit13] Ng A. M. H., Kenry, Lim C. T., Low H. Y., Loh K. P. (2014). Biosens. Bioelectron..

[cit14] Ye R., Peng Z., Wang T., Xu Y., Zhang J., Li Y., Nilewski L. G., Lin J., Tour J. M. (2015). ACS Nano.

[cit15] Lin J., Peng Z., Liu Y., Ruiz-Zepeda F., Ye R., Samuel E. L. G., Yacaman M. J., Yakobson B. I., Tour J. M. (2014). Nat. Commun..

[cit16] Prabhakaran A., Nayak P. (2019). ACS Appl. Nano Mater..

[cit17] Kurra N., Jiang Q., Nayak P., Alshareef H. N. (2019). Nano Today.

[cit18] Fenzl C., Nayak P., Hirsch T., Wolfbeis O. S., Alshareef H. N., Baeumner A. J. (2017). ACS Sens..

[cit19] Nayak P., Kurra N., Xia C., Alshareef H. N. (2016). Adv. Electron. Mater..

[cit20] Crapnell R. D., Bernalte E., Muñoz R. A. A., Banks C. E. (2025). Anal. Methods.

[cit21] Clark K. M., Nekoba D. T., Viernes K. L., Zhou J., Ray T. R. (2024). Biosens. Bioelectron..

[cit22] Nayak P., Yang M., Wang Z., Li X., Miao R., Compton R. G. (2022). Appl. Mater. Today.

[cit23] Nayak P., Kurra N., Xia C., Alshareef H. N. (2016). Adv. Electron. Mater..

[cit24] Kaniyoor A., Ramaprabhu S. (2012). AIP Adv..

[cit25] Griffiths K., Dale C., Hedley J., Kowal M. D., Kaner R. B., Keegan N. (2014). Nanoscale.

[cit26] Bird A. J. and Faulkner L. R., in Electrochemical Methods, John Wiley & Sons, New York, 2nd edn, 2010

[cit27] Rassaei L., Nebel M., Rees N. V., Compton R. G., Schuhmannb W., Marken F. (2010). Chem. Commun..

[cit28] Pankhurst J. R., Bell N. L., Zegke M., Platts L. N., Lamfsus C. A., Maron L., Natrajan L. S., Sproules S., Arnold P. L., Love J. B. (2017). Chem. Sci..

[cit29] Navarro C. B., Laker Z. P., Rourke J. P., Wilson N. R. (2015). Phys. Chem. Chem. Phys..

[cit30] Randviir E. P., Brownson D. A., Gomez-Mingot M., Kampouris D. K., Iniesta J., Banks C. E. (2012). Nanoscale.

[cit31] Huang X.-J., O'Mahony A. M., Compton R. G. (2009). Small.

[cit32] Prehn R., Abad L., Sánchez-Molas D., Duch M., Sabaté N., del Campo F. J., Muñoz F. X., Compton R. G. (2011). J. Electroanal. Chem..

[cit33] Macpherson J. V., Jones C. E., Unwin P. R. (1998). J. Phys. Chem. B.

