# Supplementary material for: Miniaturisation of a laser-scribed graphene electrode enables analyte detection at ultra-low concentrations
Source: RSC Adv. 2026 Apr 21;16(23):20629–34. doi: 10.1039/d6ra01797e (PMC13096774; doi:10.1039/d6ra01797e)
Supplement: RA-016-D6RA01797E-s001 [file RA-016-D6RA01797E-s001.pdf]

Supplementary Information (SI) for RSC Advances.
This journal is © The Royal Society of Chemistry 2026

Supporting information

Miniaturisation of laser-scribed graphene electrode enables analyte detection at ultra-low concentrations

Sarojini Prusty, Shubham B. Upadhye, Pranati Nayak*[a]
[a]*Department of Engineering and Materials Physics, Institute of Chemical Technology-Indian Oil Odisha Campus, Bhubaneswar 751013, India.*

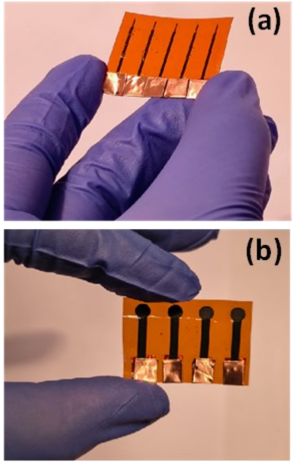

Fig. S1. Digital photograph of (a) LSG mE and (b) LSG ME arrays.

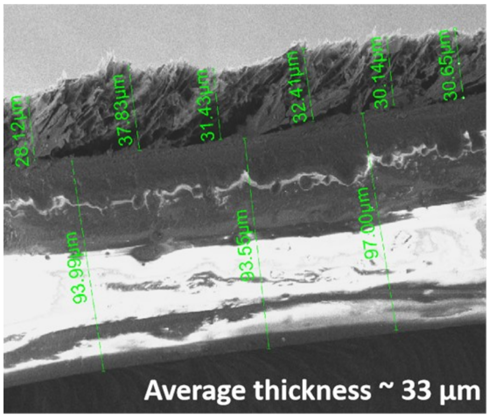

Fig. S2. Cross-sectional FESEM image of LSG electrode

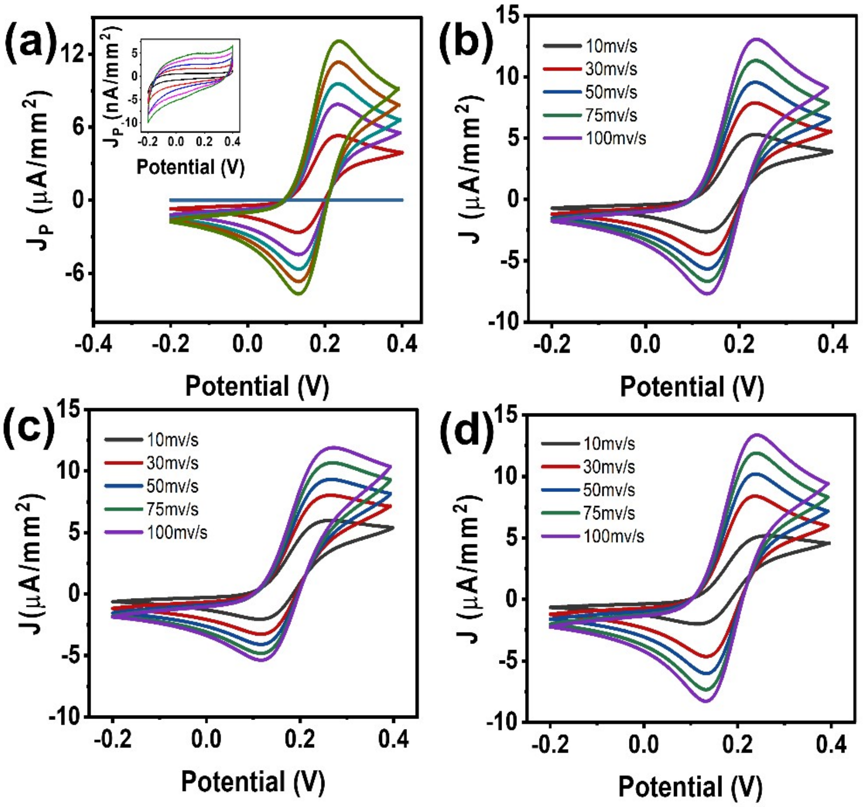

Fig. S3. Comparison CV plot of four different LSG mEs at 5mM $[(Fe(CN)_6)]^{3-/4-}$ in 0.1M KCL solution at varying scan rates starting from 10 mV/s to 100 mV/s. Inset in a show the CV response for only 0.1 M KCl solution. Inset of (a) shows plot of measured CV of LSG mEs at five different scan rates in 0.1M KCl solution.

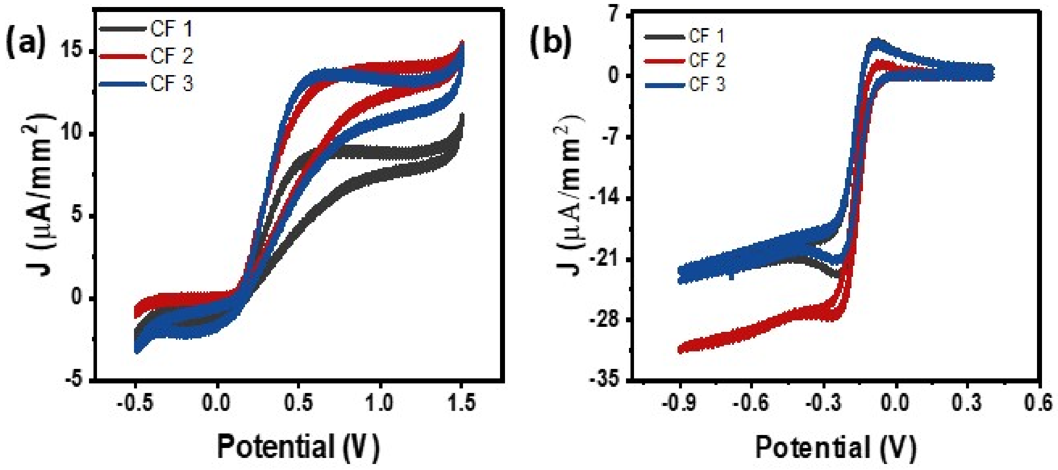

Fig. S4. Stability of 3 different CF μEs in 5mM $[(Fe(CN)_6)]^{3-/4-}$ and 5mM ($[Ru\ (NH_3)_6]^{3+/2+}$) with 5 mM KCl, Scan rate: 50 mv/s.

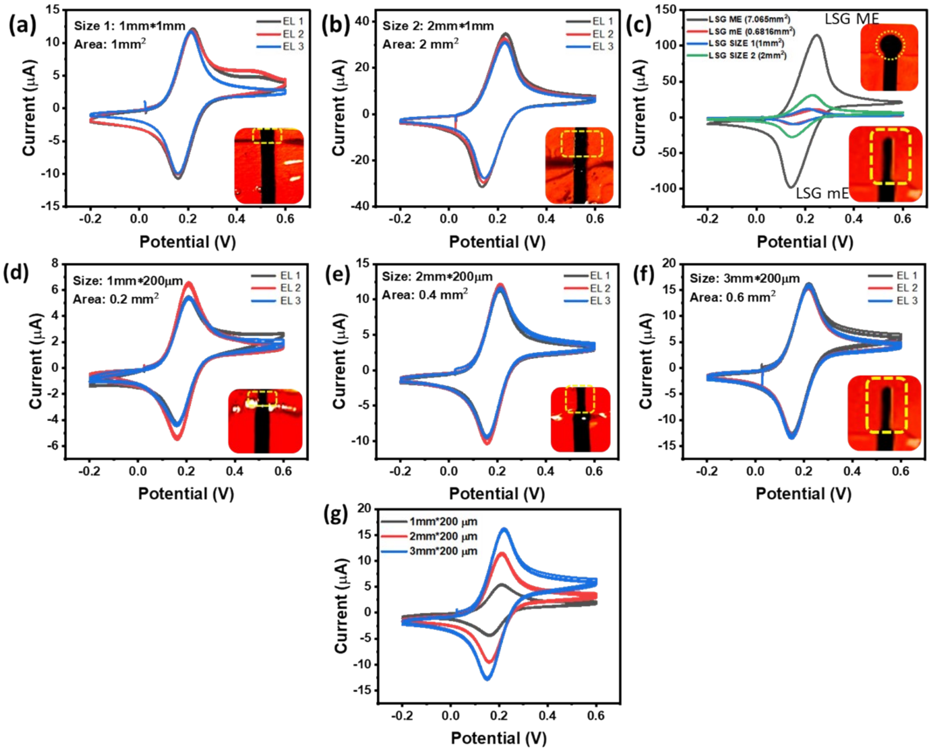

Fig. S5 CVs of LSG electrodes in 5 mM $[Fe(CN)_6]^{3-/4-}$ at 50 mV s^{-1}; Electrodes of the same type with different area (a) 1mm^2, (b) 2mm^2, and (c) their comparison with LSG mE (~0.6816 mm^2) , LSG ME (~7.065 mm^2). (d, e,and f) Electrodes of the same geometry with different sizes (0.2mm^2,0.4mm^2,0.6mm^2), (g) Comparison plot of d-f.

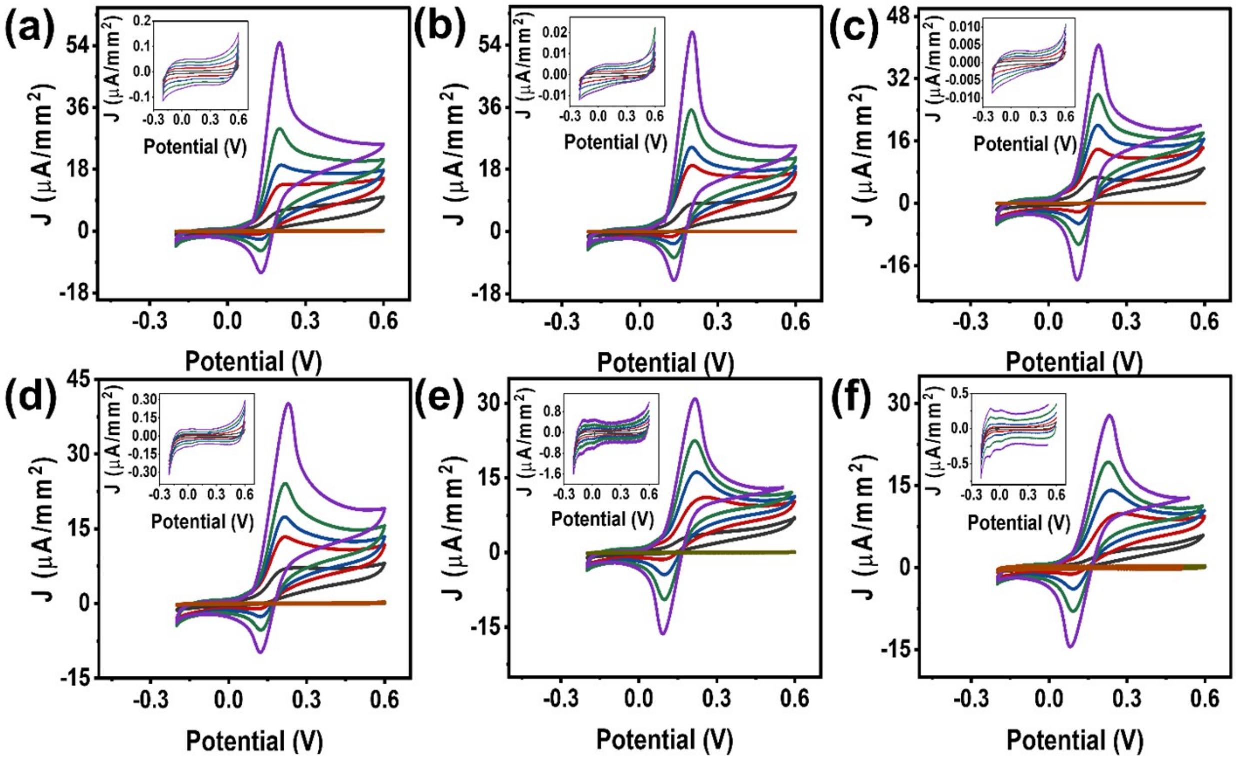

Fig. S6. Comparison CV plot of (a, b, c) 3 different LSG mEs and (d, e, f) 3 different LSG ME at different scan rate (10-100 mV/s) in 5 mM DA with 0.1 M PBS as supporting electrolyte. Insets show the plots of measured CV of LSG mEs at five different scan rate in only 0.1M PBS solution.

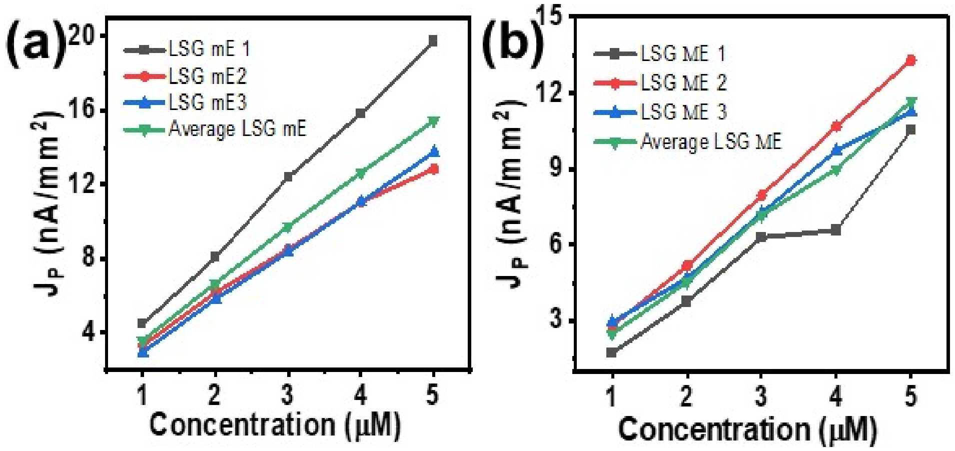

Fig. S7 Linearity plot of three different (a) LSG mEs, and (b) LSG MEs for different concentrations of Dopamine.

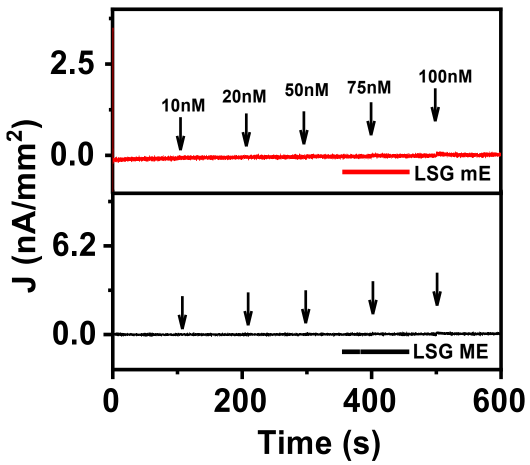

Fig. S8 Chronoamperogram for addition of ultra-low concentration of DA for LSG mE and LS
